# HPV circulating tumor DNA to monitor response to pembrolizumab and vorinostat combination in patients with advanced HPV-related squamous-cell carcinomas

**DOI:** 10.1016/j.esmoop.2025.106024

**Published:** 2025-12-29

**Authors:** D.M. Filippini, B. Cabarrou, C. Dupain, M. Halladjian, E. Coquan, M.-P. Sablin, L. Mazzarella, M. Francisco, N. Servant, M.M. Tonini, A.F. Hundt, Z. Castel-Ajgal, B. You, F. Bigot, F. Ghiringhelli, D. Vansteene, C. Gomez-Roca, S. Cousin, A. Lambert, E. Saada-Bouzid, X. Durando, C. Abdeddaim, C. Borel, R. Chaltiel, E. Borcoman, F. Legrand, S. Bernhart, M. Jimenez, I. Bièche, T. Filleron, C. Le Tourneau, M. Kamal, E. Jeannot

**Affiliations:** 1 Department of Drug Development and Innovation (D3I), Institut Curie, Paris, France; 2 Department of Medical and Surgical Sciences (DIMEC), Alma Mater Studiorum, Università di Bologna, Bologna, Italy; 3 University of Toulouse, Oncopole Claudius Regaud, IUCT-Oncopole, Biostatistics & Health Data Science Unit, Toulouse, France; 4 Department of Medical Oncology, Centre Francois Baclesse, Caen, Cedex, France; 5 Laboratory of Translational Oncology, European Institute of Oncology IRCCS, Milan, Italy; 6 Bioinformatics and Computational Systems Biology of Cancer, PSL Research University, Mines Paris Tech, INSERM U900, Paris, France; 7 Translational Medicine Operations Hub, Luxembourg Institute of Health, Luxembourg; 8 Department of Medical Oncology, Hospices de Lyon, Lyon, France; 9 Institut de Cancérologie de l'Ouest, Angers, France; 10 Centre Georges-François Leclerc, Dijon, France; 11 Institut de Cancérologie de l‘Ouest, Saint-Herblain, France; 12 Institut Claudius Regaud, IUCT-Oncopole, Toulouse, France; 13 Institut Bergonié, Bordeaux, France; 14 Institut de Cancérologie de Lorraine, Vandœuvre-Lès-Nancy, France; 15 Centre Antoine Lacassagne, Nice, France; 16 Centre Jean Perrin, Clermont-Ferrand, France; 17 Centre Oscar Lambert, Lille, France; 18 Department of Medical Oncology, ICANS, Strasbourg, France; 19 Department of Medical Oncology, Institut Jean Godinot, Reims, France; 20 R&D Department, Unicancer, Paris, France; 21 IZBI, Interdisciplinary Centre for Bioinformatics, Universität Leipzig, Leipzig, Germany; 22 Department of Genetics, Institut Curie, Paris, France; 23 Paris-Saclay University, Paris, France; 24 Department of Pathology, Institut Curie, Paris, France

**Keywords:** circulating tumor DNA, HPV, squamous cell carcinoma, biomarker, pharmacodynamic, immunotherapy

## Abstract

**Background:**

Limited data are available on the role of human papillomavirus circulating tumor DNA (HPV-ctDNA) as a pharmacodynamic marker to monitor the response to treatment in the recurrent/metastatic (R/M) setting. Our study aimed to investigate the sensitivity and pharmacodynamic value of HPV-ctDNA levels during treatment in patients with R/M HPV-related squamous cell carcinoma (SCC) treated with pembrolizumab in combination with vorinostat (PEVO trial, NCT04357873).

**Materials and methods:**

Plasma samples were prospectively collected from 57 patients with HPV-related SCC before treatment initiation and every 6 weeks until disease progression. HPV-ctDNA was quantified by droplet digital PCR. The levels before treatment were analyzed according to the patient and tumor characteristics. Landmark analyses were carried out to study the association between dynamic changes in HPV-ctDNA and progression-free survival (PFS), overall survival (OS), and overall response rate (ORR).

**Results:**

HPV-ctDNA was detected before treatment in all patients (*n* = 57) with HPV-related SCC. HPV-ctDNA levels correlated with the number of HPV copies in tumor tissue (*P* < 0.001). Higher levels of HPV-ctDNA in plasma samples were observed in anal cancer than in other tumor types (*P* < 0.001), and in patients with distant metastases with or without locoregional recurrence than in patients with locoregional recurrence alone (*P* = 0.02). The increase in HPV-ctDNA levels during treatment was associated with a lower ORR (*P* = 0.01) and shorter PFS and OS (both *P* = 0.01).

**Conclusion:**

These findings reveal that dynamic HPV-ctDNA variation levels during treatment have a pharmacodynamic value and may help monitor treatment response in patients with advanced HPV-related SCC from different locations.

## Highlights

• HPV-ctDNA was detected in all patients with HPV-related SCC before treatment.
• HPV-ctDNA levels correlated with HPV copy number in the tumor tissue.
• Pretreatment higher HPV-ctDNA levels were observed in anal cancer and metastatic disease.
• Increasing HPV-ctDNA levels during treatment were associated with poorer outcomes.

## Introduction

In an era of personalized and precision-oriented medicine, the utility of liquid biopsy is emerging to optimize therapeutic management for patients with various malignancies.^1^ Different types of circulating markers, including circulating tumor DNA (ctDNA), circulating tumor cells, microvesicles, exosomes, and microRNAs could potentially be useful for cancer screening and diagnosis, prognosis definition, and treatment response monitoring.^2^^,^^3^

In recent years, human papillomavirus circulating tumor DNA (HPV-ctDNA) has been recognized as a promising pharmacodynamic biomarker for the monitoring and surveillance of patients with HPV-related cancers.^4^, ^5^, ^6^, ^7^, ^8^ HPV-ctDNA detection using droplet digital PCR (ddPCR) has become widely established owing to its enhanced sensitivity and ability to carry out absolute quantification, surpassing the capabilities of quantitative PCR.^9^, ^10^, ^11^, ^12^ Most current evidence supporting the use of HPV-ctDNA to monitor treatment responses originated from patients undergoing chemoradiation in the curative setting for HPV-related oropharyngeal, anal, and cervical cancers.^13^, ^14^, ^15^, ^16^ Previous studies on oropharyngeal and anal cancers have shown that rapid clearance of HPV-ctDNA during treatment is associated with favorable outcomes.^13^^,^^15^ In a trial including patients with locally advanced cervical cancer, mostly squamous cell carcinoma (SCC), residual HPV-ctDNA detection after chemoradiation was associated with shorter disease-free survival and overall survival (OS).^10^ Similarly, in patients with locally advanced anal SCC, residual HPV-ctDNA after chemoradiation is associated with poor outcome.^17^

Additionally, longitudinal HPV-ctDNA analysis in patients with HPV-related tumors following curative treatment appears to be more reliable for detecting clinical disease recurrence.^18^ These findings have been observed in patients with oropharyngeal and cervical cancers, suggesting that repeated time points to draw HPV-ctDNA after primary treatment may be required to better predict relapse.^8^^,^^19^ To date, limited evidence has been documented on the clinical validity of variation in HPV-ctDNA levels as a pharmacodynamic marker of treatment response in a recurrent/metastatic (R/M) setting.^6^^,^^7^^,^^20^, ^21^, ^22^

In this study, we present the results of a longitudinal HPV-ctDNA analysis in patients with R/M HPV-related SCC from different primary locations enrolled in the PEVO trial, treated with pembrolizumab combined with vorinostat, and the correlation between dynamic changes in HPV-ctDNA and clinical outcomes.

## Materials and methods

### Study design and patients cohorts

The PEVO trial (NCT04357873) was an open-label, non-randomized, multicenter phase II basket trial that evaluated the efficacy of an immune checkpoint inhibitor targeting programmed cell death protein 1 (pembrolizumab) combined with an epidrug targeting histone deacetylases (vorinostat), in patients with R/M SCC from different primary locations.^23^ In this trial, tumor tissues before treatment initiation and sequential blood samples were collected to identify prognostic and pharmacodynamic biomarkers.^24^

**Figure 1:**
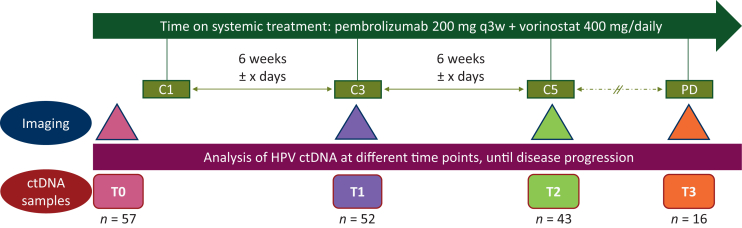
**Overview of the study design.** Number of patients selected for human papillomavirus circulating tumor DNA (HPV-ctDNA) analysis at different time points (T0 = before treatment initiation; T1 = at the first radiographic evaluation of cycle 3 of treatment; T2 = at the second radiographic evaluation of cycle 5 of treatment; T3 = at disease progression). PD, progressive disease.

Patients received only treatment with pembrolizumab 200 mg flat dose every 3 weeks administered intravenously and vorinostat 400 mg daily administered orally. Conventional computed tomography scans were carried out every 6 weeks (every two cycles). Plasma samples were collected before treatment initiation (T0) and every 6 weeks until disease progression: at cycle 3 (T1), cycle 5 (T2), and/or disease progression (T3), whichever occurred first (Figure 1). The disease staging was carried out according to the eighth edition of the American Joint Committee on Cancer.^25^ Tumor responses were evaluated according to the Response Evaluation Criteria for Solid Tumors (RECIST)1.1.^26^

This ancillary study focused on patients with R/M HPV-related SCC of the anus, cervix, vulva, vagina, penis, and head and neck. Plasma samples were collected from all patients before treatment initiation (T0) and at least one sample was collected during treatment (T1 and/or T2) or at the time of disease progression (T3).

Written informed consent was obtained from all patients before study entry. The study was compliant with the Declaration of Helsinki and Good Clinical Practice and was approved by the appropriate ethics committee. The clinical protocol was sent to French regulatory agencies in March 2020 by Unicancer and approved on 2 June 2020, by ‛Agence Nationale de Sécurité du Médicament’ and ‛Comité de Protection des Personnes’ in France, under the following references: Protocol no.: UC-GMP-1908; EudraCT no.: 2019-003839-33, and National Cancer Institute identifier: NCT04357873.

### HPV typing in tumor tissue

Total genomic DNA was isolated from tumor tissues using the AllPrep DNA/RNA Mini kit (Qiagen, Hilden, Germany) according to the manufacturer’s protocol at the Integrated BioBank of Luxembourg (IBBL). Real-time PCR using SYBR® Green PCR Master Mix and specific primers for HPV16, HPV18, and HPV33 types was carried out on a QuantStudio™ 7 Flex Real-Time PCR System (ThermoFisher Scientific, Waltham, MA), according to previously described conditions.^27^ Briefly, multiplexed amplification was carried out in a 26-μl volume using SYBR Green PCR Master Mix at a final concentration of 1×, HPV16 primers at 0.7 μM each, HPV18, and 33 primers at 1 μM each, DNA template (20 ng), and nuclease-free water.

Additional PCR using consensus GP5+/GP6+ primers, which can detect a large spectrum of HPV types, was carried out on samples found to be negative for HPV16/18/33, according to the previously described conditions.^28^ Sanger sequencing using GP5+ primer was conducted on the PCR products and HPV type was identified by comparing the obtained sequence with the reference sequences.

HPV typing was carried out on 5 μl of cell-free DNA isolated from plasma samples when tumor tissue was not available (*n* = 6).

### Plasma HPV copy number evaluation

Two PAXgene Blood circulating cell-free DNA (ccfDNA) 10-ml tubes (Cat. no. 768165 CE-IVD—stabilized specifically against cell lysis at 4°C for up to 7 days) were drawn from each participant during each collection and sent to the IBBL for immediate processing within 7 days. Plasma separation (∼4 ml from each tube) for cfDNA extraction was carried out in a two-stage centrifugation process. Separation from erythrocytes and buffy coat was achieved in the first stage by centrifugation at 1900 *g* and 4°C for 15 min. The recovered plasma was subjected to a second, high-speed (16 000 *g*, 10 min, 4°C) centrifugation to remove platelets and any remaining debris. The supernatant (3.8 ml from each of the two collection tubes per participant) were stored at –80°C until cfDNA isolation. cfDNA was manually extracted from 2 × 3.8 ml of plasma at the IBBL using the Qiagen Circulating Nucleic Acid kit (Qiagen Cat. no. 55114) on a Qiavac 24 Plus vacuum manifold (Qiagen Cat. no. 19413) following the manufacturer’s instructions. cfDNA eluates (∼30 μl in Tris-EDTA buffer) from two extractions from each participant were pooled and double-stranded DNA (dsDNA) was quantified by high-sensitivity spectrofluorometry (Quant-IT™ PicoGreen® dsDNA, ThermoFisher Cat no. P7589). The cfDNA extracts were stored at –80°C until HPV-ctDNA analysis.

HPV-ctDNA analysis was carried out by ddPCR using Taqman probes to detect the *E7* gene of the different HPV types previously identified in the tumor tissue, including HPV16, HPV18, HPV31, HPV33, HPV35, HPV59, and HPV73 types. Primer and probe sequences, amplicon sizes, and annealing temperatures are given in Supplementary Table S1, available at https://doi.org/10.1016/j.esmoop.2025.106024. The ddPCR reactions were conducted in triplicates, with the maximal volume of ctDNA template (8 μl), on the Bio-Rad QX200 ddPCR System (RRID: SCR_019707, Bio-Rad, Hercules, CA) according to the manufacturer’s protocol.^10^ The ddPCR reaction was multiplexed with a commercial human ddPCR assay targeting the *RPP30* (ribonuclease P/MRP subunit P30) gene (dHSaCP2500350, Bio-Rad Laboratories), serving as the human DNA reference, to quantify ctDNA. For each patient, DNA from the matching tumor was tested as a positive control, and a triplicate of no template DNA was tested as a negative control for each plate. Following our previous findings, plasma samples were deemed HPV positive if at least three droplets containing HPV amplicons were present.^9^ A plasma sample was considered HPV negative when fewer than three or no droplets containing HPV amplicons were detected, whereas >500 copies/ml were detected for the *RPP30* human reference gene.

### Tumor tissue HPV copy number evaluation

HPV copy number in the tumor tissue was assessed using ddPCR, with the same HPV and *RPP30* assays used in the HPV-ctDNA quantification experiment. Each experiment was carried out with 5 ng of DNA extracted from the tumor tissue. The *RRP30* gene served as a reference for diploidy, representing two copies of DNA sequences per cell. The HPV copy number in the tumor tissue was derived by doubling the ratio of HPV *E7* to *RPP30* obtained from the ddPCR experiment. This value was retained as an indication of the HPV copy number within the tumor tissue.

### Statistical analysis

This report was written in accordance with the REMARK criteria.^29^ No prespecified power was calculated because this was an ancillary study of a clinical trial. Clinicopathological characteristics and biological parameters were summarized by frequency and percentage for categorical variables and by median and range for continuous variables. Variations in HPV-ctDNA levels between T0 and T1 were calculated as the relative difference between the two time points {i.e. [HPV-ctDNA (T1) – HPV-ctDNA (T0)]/HPV-ctDNA [T0], expressed as a percentage}. A similar methodology was used to determine the variations between other time points. Associations between HPV-ctDNA levels at T0 and clinicopathological characteristics were assessed using the Mann–Whitney test. Correlation between HPV copy number in the plasma and tumor tissue was assessed using the Spearman test. Associations between variations in HPV-ctDNA level (T0-T1) and response were assessed using the Mann–Whitney test (percentage of variation) and the chi-square test (increase versus decrease).

Progression-free survival (PFS) was defined as the time from inclusion until disease progression or death from any cause, and OS as the time from inclusion to death from any cause. PFS and OS were assessed using the Kaplan–Meier method. Associations between variations in HPV-ctDNA levels (T0-T1) and PFS/OS were assessed using the log-rank test and Cox proportional hazards model. Hazard ratios (HRs) were estimated with 95% confidence interval (CI). To prevent immortal time bias, landmark survival analyses were carried out, leading to the exclusion of patients with a PFS or OS duration less than the maximum delay between inclusion and T1 (*n* = 12 and *n* = 2 patients excluded, respectively).

All statistical tests were two-sided, and *P* values < 0.05 were considered statistically significant. Statistical analyses were carried out using STATA v18 (StataCorp, College Station, TX) software.

## Results

### Patient characteristics

Among the 112 patients enrolled in the PEVO trial, we identified 65 patients (58%) with HPV-related SCC, including 57 patients (88%) with available plasma samples before treatment (T0) (Figure 1). The clinical characteristics of this HPV-ctDNA-evaluable cohort were similar to the overall PEVO trial population, with a minor difference which was a lower representation of head and neck cancers.^23^ The clinical and biological baseline characteristics according to the HPV-ctDNA levels are shown in Table 1.

**Table 1:** Patient characteristics and HPV-ctDNA levels before treatment (T0)

Characteristics	All patients, *n* (%)	HPV-ctDNA(copies/ml)		*P* value
		Median	Range	
Total	*N* = 57	1414	1-4 781 967	
Sex				0.67
Male	11 (19)	975	16-55 538	
Female	46 (81)	1415	1-4 781 967	
Age				0.40
<60 years	26 (46)	898	2-168 613	
≥60 years	31 (54)	1415	1-4 781 967	
Primary cancer site				<0.001^a^
Anus	27 (47)	18 257	25-4 781 967	
Cervix	16 (28)	234	1-39 187	
Vulva/vagina	8 (14)	1227	16-168 613	
Penis	4 (7)	520	16-1718	
Head and neck	2 (3)	27 788	38-55 538	
Disease setting				0.02^b^
LRR	8 (14)	209	13-4129	
Distant metastases (±LRR)	49 (86)	1718	1-4 781 967	0.47^c^
1 site	12 (21)	1399	2-20 654	
2 sites	20 (35)	1812	1-4 781 967	
≥3 sites	17 (30)	1763	5-342 192	
HPV types				0.07^d^
HPV16	48 (84)	1626	1-4 781 967	
Other (18, 31, 33, 35, 59, 73)	9 (16)	393	5-12 693	

*P*, Mann–Whitney test.

ctDNA, circulating tumor DNA; HPV, human papillomavirus; LRR, locoregional recurrence.

a Anus versus cervix.

b LRR versus distant.

c One site versus two or more sites.

d HPV16 versus other.

Most patients were female (81%) and aged ≥60 years (54%). The most common primary tumor location was the anus (47%), followed by the cervix (28%), vulva/vagina (14%), penis (7%), and head and neck (3%). The majority of patients (86%) had distant metastases (with or without locoregional disease) while eight patients (14%) presented with locoregional recurrence (LRR). Overall, HPV16 was detected in 84% of the tumors, whereas HPV18 was identified only in two cervical cancers. Less-frequent HPV subtypes (31, 33, 35, 59, and 73) were present in 12% of the SCC cases (Table 1). Additionally, there was no significant difference in the HPV type distribution between patients with distant metastasis (with or without locoregional disease) and patients with LRR alone (Supplementary Table S2, available at https://doi.org/10.1016/j.esmoop.2025.106024). Clinical and biological baseline features according to primary cancer site are reported in Supplementary Table S3, available at https://doi.org/10.1016/j.esmoop.2025.106024.

Out of the 57 patients included in this study, 52 (91%) had both T0 and T1 plasma samples. Among them, 43 (83%) and 15 (26%) patients had additional T2 and T3 plasma samples, respectively (Figure 1).

### HPV circulating tumor DNA detection before treatment (T0)

**Figure 2:**
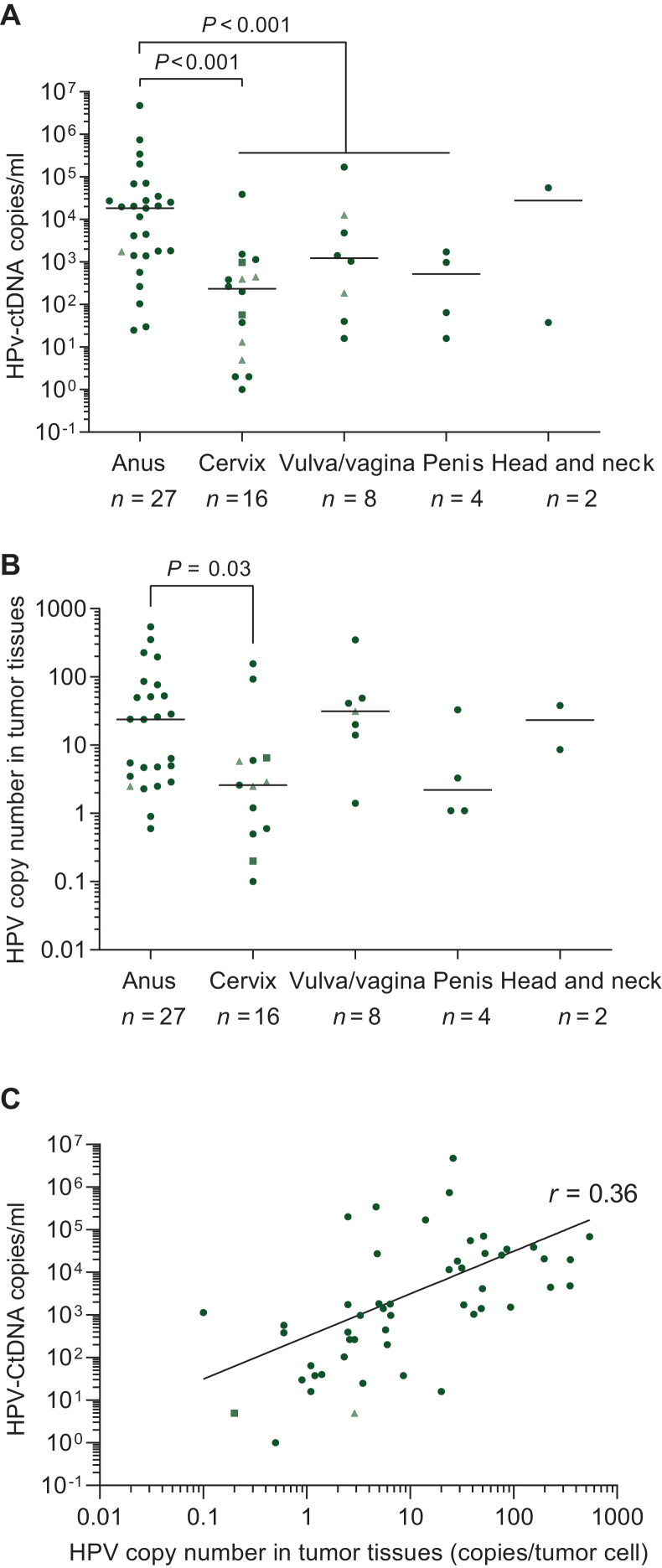
**HPV-ctDNA detection by droplet digital PCR before treatment.** Circles, squares, and triangles correspond to patients with HPV16-related tumors, HPV18-related tumors, and other HPV-related tumors, respectively. (A) HPV-ctDNA levels (log scale) according to different primary tumor locations; the group median is represented by a horizontal bar (Mann–Whitney test). (B) HPV copy number in tumor tissues (log scale) according to different primary tumor locations; the group median is represented by a horizontal bar (Mann–Whitney test). (C) Positive correlation between HPV-ctDNA levels and HPV copy number in tumor tissues (log scale). Spearman’s correlation, *r* = 0.63 (*P* < 0.001). ctDNA, circulating tumor DNA; HPV, human papillomavirus.

At T0, HPV-ctDNA was successfully detected in all 57 patients (100%) (Figure 2A). The median HPV-ctDNA concentration at T0 was 1414 copies/ml (range 1-4 781 967)] in the entire cohort (Table 1). There was no significant association between HPV-ctDNA levels and age, sex, or HPV type (Table 1).

We observed a significant difference in HPV-ctDNA levels according to primary tumor location. HPV-ctDNA levels were significantly higher in the anal cohort than in the cervical cohort (*P* < 0.001) (Table 1, Figure 2A). This difference was still significant when comparing anal cancers with all genital cancers combined (cervical, penile, vulvar, and vaginal tumors) (*P* < 0.001) (Figure 2A). This significant difference was still observed when the analysis was carried out with only HPV16-related cancers (Supplementary Figure S1, available at https://doi.org/10.1016/j.esmoop.2025.106024). Head and neck squamous cell carcinoma (HNSCC) was excluded from the analysis owing to its limited sample size (*n* = 2). Similarly, the HPV copy number in tumor tissue was significantly higher in the anal cancer cohort than in the cervical cancer cohort (*P* = 0.03) (Figure 2B). Based on the analysis of 51 patients with available tumor tissue, we observed a positive correlation between plasma HPV-ctDNA levels at T0 and HPV copy number in the tumor tissue (*r* = 0.63, *P* < 0.001, Spearman) (Figure 2C).

Finally, we observed that the median level of HPV-ctDNA at T0, in patients with distant metastases with or without locoregional disease, was significantly higher than that in patients with locoregional recurrence alone (1718 copies/ml versus 209 copies/ml, *P* = 0.02) (Table 1). However, this difference in level was not significantly associated with the number of distant metastatic sites (Table 1).

### Pharmacodynamic value of HPV-ctDNA levels

Out of 57 patients, 52 were assessable for the evaluation of HPV-ctDNA level variations between T0 and T1. All patients experienced an increase or decrease in HPV-ctDNA levels (range of variation –100% to +5700%). Among them, 19 patients (36%) demonstrated an objective response to treatment, including 7 patients (13%) with a complete response (CR) and 12 patients (23%) with a partial response (PR). Stable disease (SD) was observed in 21 patients (40%), while 12 patients (23%) experienced an upfront progressive disease (PD) (Supplementary Table S4, available at https://doi.org/10.1016/j.esmoop.2025.106024).

**Figure 3:**
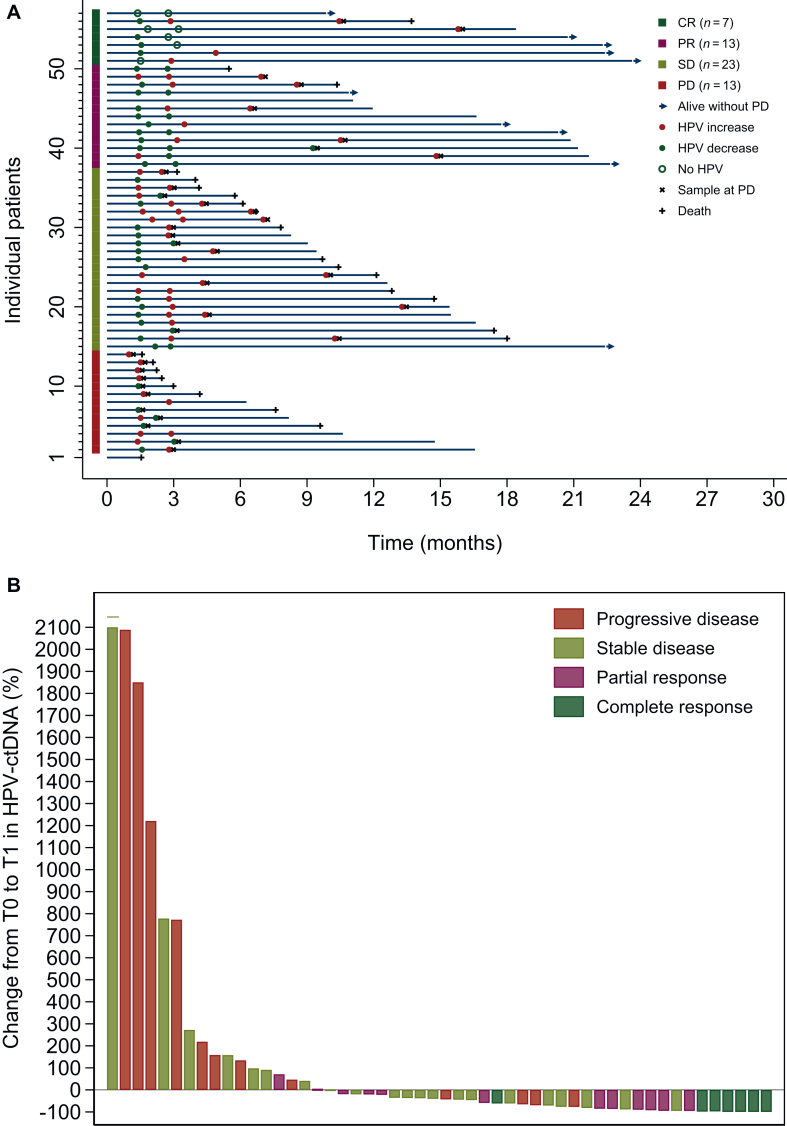
**Evolution of HPV-ctDNA during treatment.** (A) Each line represents a single patient (*n* = 57). The length of each line corresponds to the duration of follow-up. Red (•), green (•), and white (•) circles correspond to an increase in HPV-ctDNA level, a decrease in HPV-ctDNA level, and clearance of HPV-ctDNA, respectively. Time zero indicated the time of enrollment in the study. Blue arrow (→), black cross (x), and plus sign (+) indicate patients alive without PD, disease progression, and death, respectively. (B) Waterfall plot illustrating response according to HPV-ctDNA fold change levels (%) from T0 to T1 in each patient (*n* = 52). For one patient, the fold change was 5700% and was represented as a fold change of 2100% with an upper horizontal line (-) for graphical reasons. CR, complete response; ctDNA, circulating tumor DNA; HPV, human papillomavirus; PD, progressive disease; PR, partial response; SD, stable disease.

All patients who experienced CR had a decrease (*n* = 4) or complete clearance (*n* = 3) of HPV-ctDNA levels in their T1 samples compared with T0 (Figure 3A). Clearance did not seem to be linked to lower HPV-ctDNA levels at T0 (data not shown). In 10 out of the 12 patients (83%) who achieved a PR and with an available T1 sample, a decrease in HPV-ctDNA levels at T1 was also observed (Figure 3A). Overall, we observed that the early decrease in HPV-ctDNA levels at T1 was significantly more frequent in responder patients (CR/PR) than in non-responder patients (SD/PD) (89.5% versus 54.5%, *P* = 0.01), and the level of decrease was significantly higher in responder patients (*P* < 0.001) (Figure 3B, Supplementary Table S4, available at https://doi.org/10.1016/j.esmoop.2025.106024). Conversely, among the non-responder patients, we observed that the six highest increases in HPV-ctDNA levels at T1 (change >500%) (Figure 3B) did not seem to be associated with a more rapid progression or a worse prognosis (patients #6, #9, #12, #14, #34, and #37), and their outcome was comparable to the other non-responders (Figure 3A).

At T2, we observed an increase in HPV-ctDNA levels in almost all non-responder patients (18/24, 75%), while we observed a clearance or a decrease in HPV-ctDNA levels in more than half of the responder patients (11/19, 58%) (Figure 3A). Among the responder patients with a clearance or a decrease in HPV-ctDNA at T2, almost all patients (8/11, 75%) were still alive without PD after 10 months of treatment (Figure 3A).

### Prognostic value of increasing HPV-ctDNA levels at T1 on PFS and OS outcomes

**Figure 4:**
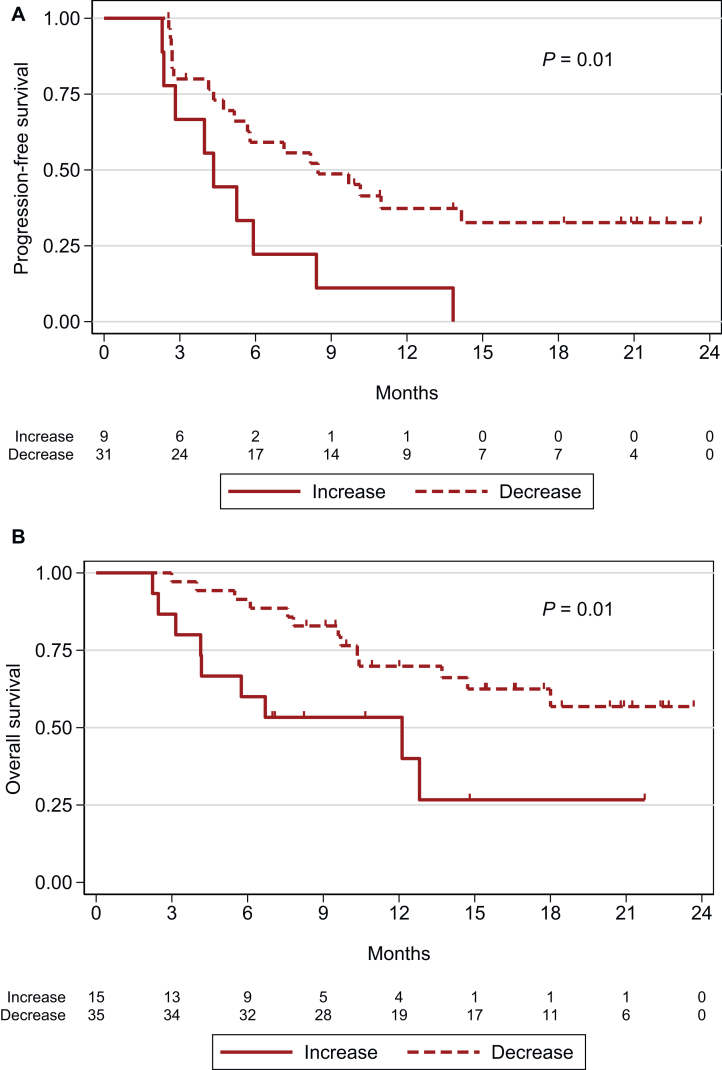
**Survival analyses according to HPV-ctDNA variation levels between T0 and T1 across all cohorts.** (A) Progression-free survival (*n* = 40). (B) Overall survival (*n* = 50). ctDNA, circulating tumor DNA; HPV, human papillomavirus.

Increasing HPV-ctDNA levels from T0 to T1 were strongly correlated with PFS and OS. The 6-month PFS rate was 22.2% (95% CI 3.4% to 51.3%) in patients with increasing HPV-ctDNA levels, and 59.1% (95% CI 39.3% to 74.4%) in patients with decreasing HPV-ctDNA levels (*P* = 0.01) (Figure 4A). Similarly, the 6-month OS rate was 60% (95% CI 31.8% to 79.7%) in patients with increasing T0-T1 HPV-ctDNA levels versus 91.4% (95% CI 75.7% to 97.2%) in patients with decreasing HPV-ctDNA levels (*P* = 0.01) (Figure 4B).

Taken as a continuous variable, an increase in HPV-ctDNA between T0 and T1 was associated with shorter PFS [HR (per 10% variation unit) = 1.01, 95% CI 1.00-1.01, *P* = 0.004] and OS [HR (per 10% variation unit) = 1.00, 95% CI 1.00-1.01, *P* = 0.005] in univariable analysis. No multivariable analyses for OS and PFS were carried out because of the small number of patients.

## Discussion

In this prospective ancillary study of a phase II basket trial involving different cohorts of patients with R/M HPV-related SCC, we observed that (i) HPV-ctDNA was detected before treatment in all patients and (ii) variations in HPV-ctDNA levels during treatment predicted tumor response and survival.

The detection of HPV-ctDNA by ddPCR is feasible and reliable as it reflects the abundance of circulating copies. Compared with other methods, ddPCR is a rapid, cost-effective, and highly sensitive technique that offers enhanced sensitivity, reproducibility, and absolute quantification for HPV-ctDNA detection.^9^ Our 100% detection rate of HPV-ctDNA by ddPCR in pretreatment blood samples is in line with previous studies in both localized and R/M HPV-related cancer types.^6^^,^^30^ HPV-ctDNA levels correlated with HPV copy number in tumor tissue, consistent with our previous findings.^10^^,^^19^ It remains to establish a cut-off value for pretreatment HPV-ctDNA levels and investigate its prognostic significance, similar to the approaches taken by Chung et al. and Mazurek et al., who recently reported that patients with R/M and localized disease, respectively, presenting pretreatment high levels of HPV-ctDNA had a lower overall response rate, shorter PFS/OS, and improved risk of distant metastases.^21^^,^^31^

Our results also suggest that, beyond the viral load in the tumor tissue, several factors such as disease setting (locoregional recurrence alone versus metastatic disease with or without locoregional recurrence) and primary tumor site may influence HPV-ctDNA level detection. Previous studies, mainly in localized/locally advanced cervical cancer, reported that levels of HPV-ctDNA increased with tumor stage, and that the HPV detection rate was also significantly higher in patients with lymph node involvement.^10^^,^^19^^,^^32^, ^33^, ^34^ In our study, patients with metastatic disease with or without locoregional recurrence exhibited higher levels of HPV-ctDNA than those with only locoregional recurrence, supporting the correlation between tumor burden and ctDNA levels, as reported in other studies.^22^^,^^35^, ^36^, ^37^ The specific relationship between plasma biomarker levels and tumor burden is complex and may be influenced by factors such as greater apoptosis rates in metastatic disease with a subsequent release of more ctDNA into the bloodstream than localized recurrence.^35^

We report for the first time that HPV-ctDNA levels may differ according to cancer type since HPV-ctDNA levels were significantly higher in anal cancer than in cervical cancer or all genital cancers taken together. This difference might be explained by the variation in HPV copy number in the tumor tissue, with anal cancers presenting the highest tumoral HPV copy number levels. Further studies are needed to confirm this result and investigate whether additional factors, such as HPV type, may have an impact on HPV-ctDNA levels. Due to the predominance of HPV16 in our cohort, we were unable to establish a link between other HPV types in the tumor tissue and ctDNA levels. Indeed, previous studies have reported either lower or higher detection rate of HPV-ctDNA in HPV18-related tumors.^10^, ^11^, ^12^^,^^38^

Finally, the main result of our study was the pharmacodynamic value of HPV-ctDNA levels during treatment in patients with advanced SCC from different primary sites. Increased HPV-ctDNA levels during treatment were significantly associated with a lack of objective response and poor survival. All patients with CR consistently demonstrated either a decrease or complete clearance, whereas the interpretation of HPV-ctDNA levels in patients who achieved a PR, SD, or PD was more complex. This complexity may be related to limitations in the RECIST criteria, such as the variability in tumor measurements, bias in selecting target lesions, heterogeneity within lesions, and the possibility of pseudoprogression.^39^

To date, few studies, evaluating the kinetics of HPV-ctDNA and its correlation with clinical outcomes, have been conducted in small cohorts of patients with R/M HNSCC or cervical/vulvar cancers treated with immunotherapy.^6^^,^^20^^,^^22^ Although our study involved a combination of immune-based treatments, including pembrolizumab and vorinostat, our findings are consistent with those of previous studies involving immunotherapy. Kang et al. reported that, in a cohort of nine patients with metastatic cervical cancer receiving tumor-infiltrating lymphocyte infusion, six patients who had PD remained positive for HPV-ctDNA at all time points, whereas the three responder patients turned negative for HPV-ctDNA early after treatment initiation.^6^ Haring et al. also observed that an increase of >60% in HPV-ctDNA levels after one cycle of treatment was associated with PD in a cohort of 16 R/M HNSCC patients, including seven patients undergoing immunotherapy.^20^ In a report of an R/M anal SCC patient who experienced PR to single-agent nivolumab, a transient 10% increase in HPV-ctDNA immediately after the second dose of nivolumab was documented, after which HPV-ctDNA dropped by >95% after four doses.^7^ More recently, Huffman et al. reported a significant correlation between decreasing levels of HPV-ctDNA from baseline to 3 and 6 weeks and clinical benefit (defined as the percentage of patients with a CR, PR, or SD for at least 6 months) and longer PFS compared with patients with increasing levels during treatment with pembrolizumab, in a cohort of 32 patients with metastatic or locally advanced incurable anal SCC.^40^

Although not tested in this study, HPV-ctDNA could help clinicians interpret equivocal imaging results, such as pseudoprogression or mixed responses to immune checkpoint inhibitors.^20^^,^^41^^,^^42^

We acknowledge that our study has some limitations. Firstly, ctDNA analysis was carried out in ∼50% of the patients, reflecting that about half of the PEVO study population had HPV-related tumors with available blood samples suitable for HPV-ctDNA assessment. In addition, some cohorts, such as those with HNSCC and penile cancer, had few patients, which may restrict the generalizability of our findings. Secondly, most tumors were HPV16-related, with a limited representation of HPV18 and other less common HPV types. Consequently, we were unable to observe an association between HPV-ctDNA levels and any specific HPV type. Moreover, in the present study, all tumors were HPV-related squamous-cell carcinoma, the main histological type of HPV-related tumors. The results remain to be confirmed in other histological types of HPV-related tumors. Finally, nodal burden was not evaluated in our study, so we could not investigate whether the nodal stage of the tumor was associated with increased HPV-ctDNA levels, as previously reported in the literature.^10^^,^^19^^,^^31^^,^^43^

In conclusion, our study confirms that the HPV *E7* gene is a sensitive and suitable pharmacodynamic biomarker for monitoring the response to treatment of patients with R/M HPV-related SCC, undergoing novel therapeutic combination strategies (pembrolizumab and vorinostat). Further randomized studies are warranted to validate the clinical implications of HPV-ctDNA levels and should establish whether a threshold for HPV-ctDNA variation during treatment may guide clinical decision making. This would be of specific interest for evaluating the feasibility of therapy discontinuation in long-term responders following clearance and for predicting the need to resume therapy when required.
