# Supplementary material for: HPV circulating tumor DNA to monitor response to pembrolizumab and vorinostat combination in patients with advanced HPV-related squamous-cell carcinomas
Source: ESMO Open. 2025 Dec 29;11(1):106024. doi: 10.1016/j.esmoop.2025.106024 (PMC12804039; doi:10.1016/j.esmoop.2025.106024)
Supplement: Supplementary Figure S1 [file mmc1.pptx]

## Slide 1
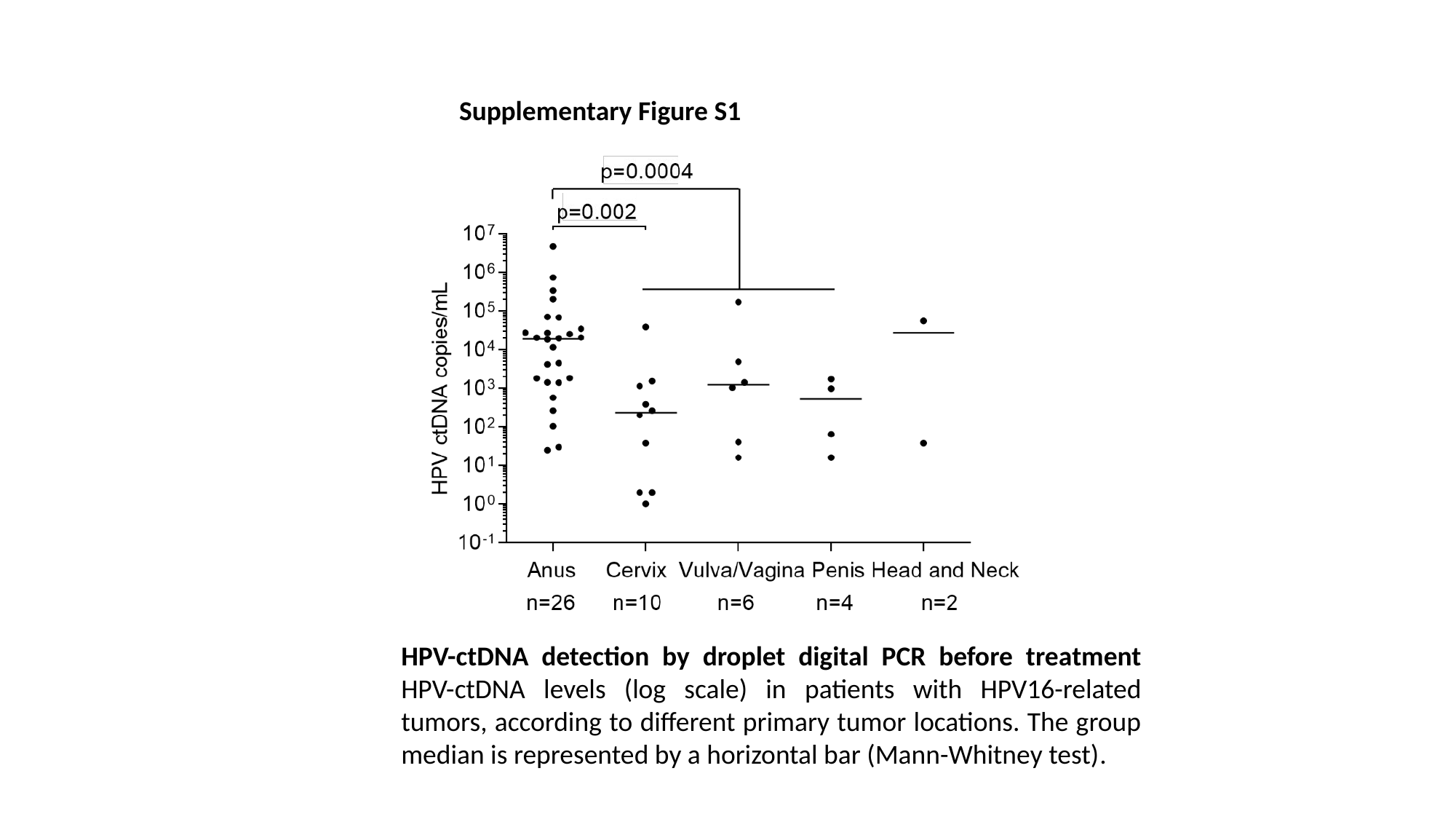

Supplementary Figure S1
HPV-ctDNA detection by droplet digital PCR before treatment HPV-ctDNA levels (log scale) in patients with HPV16-related tumors, according to different primary tumor locations. The group median is represented by a horizontal bar (Mann-Whitney test).
