# Supplementary material for: HPV circulating tumor DNA to monitor response to pembrolizumab and vorinostat combination in patients with advanced HPV-related squamous-cell carcinomas
Source: ESMO Open. 2025 Dec 29;11(1):106024. doi: 10.1016/j.esmoop.2025.106024 (PMC12804039; doi:10.1016/j.esmoop.2025.106024)
Supplement: Supplementary Tables S1 to S4 [file mmc2.docx]

**Supplementary Table S1:** **Primer and probe sequences, amplicon sizes and annealing temperatures**

| HPV type | Forward sequence | Reverse sequence | Probe sequence | Amplicon size | Annealing temperature |
| --- | --- | --- | --- | --- | --- |
| HPV16 | TCCAGCTGGACAAGCAGAAC | CACAACCGAAGCGTAGAGTC | ACAGAGCCCATTACAAT-6FAM | 88 pb | 60°C |
| HPV18 | AACATTTACCAGCCCGACGA | TCGTCTGCTGAGCTTTCTAC | AACCACAACGTCACACAA-6FAM | 106 pb | 60°C |
| HPV31 | CGTTACCTTTTGTTGTCAGTGT | GAACAGTTGGGGCACACGA | ACAGAGCACACAAGTAG-6FAM | 123 pb | 56°C |
| HPV33 | CAGATGAGGATGAAGGCTTGGA | ACTGTTGACACATAAACGAACTG | CTTGTCCATCTGGCC-6FAM | 119 pb | 56°C |
| HPV35 | AGGCGACACTACGTCTGT | ACACTATTCCAAATGTGCCCA | ACAGAGCACACACATTG-6FAM | 87 pb | 56°C |
| HPV59 | CAATTACCTGACTCCGACTCC | ATGTTGTGACGCTGTGGTTC | AGTAGCAAAGGATGATTAAC-6FAM | 101 pb | 56°C |
| HPV73 | TCAATGGCAAGGCATACTGTG | AGGATGAGGATGAAACAGACAGC | ACTATTCTGTAACACTCTCGT-6FAM | 109 pb | 56°C |

**Supplementary Table S2: HPV type according to the disease status at inclusion**

|  | Total | Distant metastases (+/-LRR) | LRR |  |
| --- | --- | --- | --- | --- |
| HPV types | N=57 | N=49 | N=8 |  |
| HPV16 | 48 (84%) | 42 (86%) | 6 (75%) |  |
| HPV18 | 2 (3.5%) | 2 (4%) | 0 (0%) |  |
| HPV31 | 1 (1.8%) | 1 (2%) | 0 (0%) |  |
| HPV33 | 1 (1.8%) | 1 (2%) | 0 (0%) |  |
| HPV35 | 1 (1.8%) | 0 (0%) | 1 (12.5%) |  |
| HPV59 | 2 (3.5%) | 1 (2%) | 1 (12.5%) |  |
| HPV73 | 2 (3.5%) | 2 (4%) | 0 (0%) |  |
|  |  |  |  | *p-value* |
| HPV16 | 48 (84%) | 42 (86%) | 6 (75%) | 0.6 |
| Other HPV | 9 (16%) | 7 (14%) | 2 (25%) |  |

*Abbreviations: LRR: Locoregional recurrence; p: Fisher test.*

**Supplemental Table S3: Patient characteristics according to the primary cancer site**

|  |  | **Primary cancer site** | | | | | |  |
| --- | --- | --- | --- | --- | --- | --- | --- | --- |
|  | **Total** | | **Anus** | **Cervix** | **Vulva/Vagina** | **Penis** | **Head and Neck** | |
|  | (n = 57) | | (n = 27) | (n = 16) | (n = 8) | (n = 4) | (n = 2) | |
| **Sex** |  | |  |  |  |  |  | |
| Male | 11 (19%) | | 5 (18%) | - | - | 4 (100%) | 2 (100%) | |
| Female | 46 (81%) | | 22 (81%) | 16 (100%) | 8 (100%) | - | 0 (0%) | |
| **Age** |  | |  |  |  |  |  | |
| <60 years | 26 (46%) | | 11 (41%) | 10 (62%) | 3 (37%) | 0 (0%) | 2 (100%) | |
| >60 years | 31 (54%) | | 16 (59%) | 6 (37%) | 5 (62%) | 4 (100%) | 0 (0%) | |
| **Disease setting** |  | |  |  |  |  |  | |
| Distant | 49 (86%) | | 25 (93%) | 12 (75%) | 7 (87%) | 3 (75%) | 2 (100%) | |
| metastases (+/-LRR) |  | |  |  |  |  |  | |
| 1 site | 12 (24%) | | 6 (24%) | 3 (25%) | 2 (29%) | 1 (33%) | 0 (0%) | |
| 2 sites | 20 (41%) | | 10(40%) | 5 (42%) | 2 (29%) | 2 (67%) | 1 (50%) | |
| ≥3 sites | 17 (35%) | | 9 (36%) | 4 (33%) | 3 (43%) | 0 (0%) | 1 (50%) | |
| LRR only | 8 (14%) | | 2 (7%) | 4 (25%) | 1 (12%) | 1 (25%) | 0 (0%) | |
| **HPV types** |  | |  |  |  |  |  | |
| HPV16 | 48 (84.5%) | | 26 (96%) | 10 (63%) | 6 (75%) | 4 (100%) | 2 (100%) | |
| HPV18 | 2 (3.5%) | | 0 (0%) | 2 (12.5%) | 0 (0%) | 0 (0%) | 0 (0%) | |
| HPV31 | 1 (2%) | | 1 (4%) | 0 (0%) | 0 (0%) | 0 (0%) | 0 (0%) | |
| HPV33 | 1 (2%) | | 0 (0%) | 0 (0%) | 1 (12.5%) | 0 (0%) | 0 (0%) | |
| HPV35 | 1 (2%) | | 0 (0%) | 1 (6%) | 0 (0%) | 0 (0%) | 0 (0%) | |
| HPV59 | 2 (3.5%) | | 0 (0%) | 2 (12.5%) | 0 (0%) | 0 (0%) | 0 (0%) | |
| HPV73 | 2 (3.5%) | | 0 (0%) | 1 (6%) | 1 (12.5%) | 0 (0%) | 0 (0%) | |
| **Best response** |  | |  |  |  |  |  | |
| CR | 7(12%) | | 4 (15%) | 2 (12%) | 1 (12%) | 0 (0%) | 0 (0%) | |
| PR | 13(23%) | | 5 (19%) | 5 (31%) | 2 (25%) | 1 (25%) | 0 (0%) | |
| SD | 23(40%) | | 12 (44%) | 6 (38%) | 0 (0%) | 3 (75%) | 2 (100%) | |
| PD | 13(23%) | | 5 (19%) | 3 (19%) | 5 (62%) | 0 (0%) | 0 (0%) | |
| NA | 1(2%) | | 1 (4%) | 0 (0%) | 0 (0%) | 0 (0%) | 0 (0%) | |

*Abbreviations : LRR: locoregional recurrence; CR: complete response; PR: partial response ; SD: stable disease; PD: progressive disease; NA: not assessed.*

**Supplemental Table S4: Association between HPV-ctDNA levels change between T0 and T1 and best overall response**

|  | **Total**  (N = 52) | **CR**  (N = 7) | **PR**  (N = 12) | **SD**  (N = 21) | **PD**  (N = 12) | ***p-value***  ***^(CR/PR vs SD/PD)^*** |
| --- | --- | --- | --- | --- | --- | --- |
| **HPV-ctDNA variation (%)** |  |  |  |  |  | *<0.001* |
| Median | -42.1 | -100 | -73 | -37 | 146 |  |
| (Range) | (-100; 5,700) | (-100; -61) | (-96; 71) | (-96; 5,700) | (-77; 2,100) |  |
| **HPV-ctDNA level change** |  |  |  |  |  | *0.01* |
| Increase | 17 (33%) | 0 (0%) | 2 (17%) | 7 (33%) | 8 (67%) |  |
| Decrease | 35 (67%) | 7 (100%) | 10 (83%) | 14 (67%) | 4 (33%) |  |

Abbreviations: CR, complete response; PR, partial response ; SD, stable disease; PD, progressive disease; HPV: Human Papillomavirus; T0, before treatment initiation; T1, at cycle three of treatment.
